# Smoking Trends among Thailand’s Youths from 1996–2015: An Age-Period-Cohort Analysis of National Health Surveys

**Published:** 2019-03

**Authors:** Nirun INTARUT, Rassamee SANGTHONG, Virasakdi CHONGSUVIVATWONG

**Affiliations:** 1. Faculty of Medicine, Mahasarakham University, Muang, Mahasarakham, Thailand; 2. Clinical Epidemiology Unit, Faculty of Medicine, Mahasarakham University, Muang, Mahasrakham, Thailand; 3. Epidemiology Unit, Faculty of Medicine, Prince of Songkla University, Hat Yai, Songkhla, Thailand

**Keywords:** Secular trend, Smoking trend, Youth smoking, Age-period-cohort model

## Abstract

**Background:**

This study aimed to investigate secular trends of smoking among Thailand’s youths.

**Methods:**

We combined 8 datasets from national representative surveys between 1996 and 2015. Multi-stage cluster sampling was applied in all studies. Overall, 231459 participants aged 11–26 yr were included and analyzed. Participants were classified as current smokers if they responded “yes” to the question “Do you currently smoke?”, and former smoker if they reported no current smoking but had smoked previously. Age-period-cohort (APC) models were used to estimate age, period, and cohort effects on smoking for investigating secular trend of smoking.

**Results:**

The prevalence of smoking tended to decrease over time. Among those aged 11–14, the prevalence of current and former smoking was low but not negligible. Rates of underage smoking remained quite steady, around 3.8% in 1996 and 3.6% in 2015. The results of the APC model show that the prevalence of smoking among young male cohorts was lower than in older cohorts.

**Conclusion:**

Thailand’s tobacco control program has been effective in deterring youths from smoking. The prevalence of smoking in this population needs to be reduced further though, something achieved by reorienting tobacco consumption prevention campaigns towards this age group.

## Introduction

In accordance with the World Health Organization Framework Convention on Tobacco Control (WHO FCTC), Thailand has been implementing tobacco control policies since 1970 (1, 2). One of the priorities of these policies is to prevent young populations from consuming tobacco products (3, 4), thereby preventing or reducing addiction later in life (5). As recently as 2013, the prevalence of smoking among young Thai males (aged 13–15 yr) was 20.1% (6), a value considered too high for this age group.

Youths are vulnerable to smoking due to their inquisitive nature and are thus a lucrative target for tobacco industries (7, 8). Youths progressively increase the frequency with which they smoke due to the addictive nature of nicotine, even though initially they may only intermittently consume these products (9–11). A decrease in the age at which people start smoking and an increase in duration of tobacco consumption provide great benefits to industries whilst causing substantially poorer health for consumers.

Most Thai youths have been exposed to antismoking campaigns since they were children. Epidemiologically, the trends of smoking prevalence can be explained by three inter-related effects: age, period, and cohort (12, 13). The age effect is the variation associated with growing old and is quantified by the prevalence of smoking with age after controlling for period and cohort. The period effect describes changes in cultural, social or economic circumstances at different points in time. The cohort effect describes the effect of different generations that move through life together, encountering the same historical and social events at the same ages.

Since the tobacco consumption policy measures were employed, younger had passed thought from youth, adolescent and young adult simultaneous with those measures on them. Now, it has limited a study aimed to investigate the trends of smoking in adolescent in Thailand. In order to develop or improve tobacco and alcohol control measures, it is necessary to examine the trends in smoking among youth as they may reflect the effectiveness on this national control programs. There were several births cohort involved and their aged over the time period, analysis takes their simultaneous effects will bring better understanding.

It is essential to understand the trends of tobacco consumption among youths in order to implement age-appropriate control measures (14). Therefore, this study investigated secular trends in smoking using eight national surveys conducted between 1999 and 2015 using age-period-cohort analysis.

## Materials and Methods

### Study sample

Data from eight national surveys were obtained from Thailand’s National Statistical Office. These included the five most recent Health and Welfare Surveys conducted in 1996, 2001, 2009, 2013, and 2015, and three Smoking and Drinking Behavior Surveys conducted in 2004, 2007, and 2011. All surveys used a stratified two-stage sampling technique for collecting nationally representative data. All provinces were included in the survey, and each province was stratified by area of residence (in- and outside the municipality area) (15). A systematic random sampling technique was applied for selecting villages in each residential area. Households in each selected village were randomly selected using simple random sampling. Only the head of the household was interviewed in the Health and Welfare Surveys, but all household members were interviewed in the Smoking and Drinking Behavior Surveys. Face-to-face interviews were conducted using a structured-questionnaire by a well-trained interviewer and all interviews were conducted in a private manner.

Baseline characteristics included age, gender, years of school attendance, marital status (single, married, or other) and area of residence (urban or rural area). Participants were classified as current smokers if they responded “yes” to the question “Do you currently smoke?” Participants were classified as former smokers if they reported no current smoking but had smoked previously. Former smoking was computed using current and former smokers as the denominator.

### Statistical analysis

Age was grouped into four 3-year intervals (11–14, 15–18, 19–22 and 23–26). Period was identified by the study survey year (1996, 2001, 2004, 2007, 2009, 2011, 2013, and 2015). Birth cohorts were calculated as the study period relative to age at survey (1969–1972, 1973–1976, 1977–1980, 1981–1984, 1985–1988, 1989–1992, 1993–1996, and 1997–2000).

Age-period-cohort (APC) analysis was used for estimating age, period, and cohort effects (13, 16, 17). Because these three effects are interrelated, it is difficult to distinguish them from each other. This is known as the identifiability problem. To overcome this, we used the constraint method (16, 18). For estimating the effects, this model can be written as (16):

$$\mathrm{Log}(Y_{ij})=\text{log}(P_{ij})+\mu +\alpha_{i}(\mathrm{Age})+\beta_{j}(\mathrm{Period})+\gamma_{k}(\mathrm{Cohort})$$

where Y_ij_ denotes the observed rate of smoking in the *i*^th^ age group, and the *j*^th^ period (*j*^th^); *P*_*ij*_ represents the size of the estimated population in the *ij*^th^ age/period group, μ represents the intercept; ∝_*i*_ denotes the effect for the *i*^th^ age group; *β*_*j*_ denotes the effect for the *j*^th^ time period; *γ*_*κ*_ denotes the effect for the *k*^th^ cohort.

The coefficient of prevalence rate for the age, period, and cohort effects were presented together with 95% confidence intervals. All data analysis was performed by R version 2.15.3 using Epi and epicalc (19, 20).

## Results

Overall, 231459 participants from surveys performed between 1996 and 2015 were included in this study. Table 1 shows the characteristics of the study sample. Males and females were distributed equally throughout all surveys. Most had attended 1–6 yr of school. More than three-quarters of participants were single and more than half lived in urban areas. The prevalence of smoking declined from 13.18% in 1996 to 10.94% in 2015. However, between 2009 and 2015 the prevalence of smoking was slightly higher than in previous years. In the underage group (i.e. aged less than 18 yr), underage smoking was 3.8% in 1996 and 3.6% in 2015.

**Table 1:** Characteristics of the study samples between 1996 and 2015. Values presented outside parentheses represent numbers, whilst those inside parentheses represent percentages

***Characteristics***	***Survey year and number (n) of participants***								***Total***
	***1996***	***2001***	***2004***	***2007***	***2009***	***2011***	***2013***	***2015***	
	***n=21,958***	***n=54,329***	***n=15,556***	***n=46,907***	***n=15,673***	***n=36,358***	***n=14,163***	***n=26,515***	***n=231,459***
Prevalence of smoking	13.2%	11.0%	10.0%	9.3%	9.9%	10.7%	10.9%	10.9%	10.6%
Prevalence of underage smoking (<18 yr old)	3.8%	2.8%	2.8%	3.1%	3.1%	3.8%	3.9%	3.6%	3.3%
Gender									
Male	10588 (48.2)	26631 (49.0)	7684 (49.4)	23191 (49.4)	7855 (50.1)	18326 (50.4)	7129 (50.3)	13283 (50.1)	114687 (49.5)
Female	11370 (51.8)	27698 (51.0)	7872 (50.6)	23716 (50.6)	7818 (49.9)	18032 (49.6)	7034 (49.7)	13232 (49.9)	116772 (50.5)
Age (years)									
11–14	6556 (29.9)	15659 (28.8)	5050 (32.5)	16035 (34.2)	5223 (33.3)	11172 (30.7)	4090 (28.9)	7660 (28.9)	71445 (30.9)
15–18	5583 (25.4)	14458 (26.6)	3936 (25.3)	12903 (27.5)	4318 (27.6)	10369 (28.5)	4224 (29.8)	7714 (29.1)	63505 (27.4)
19–22	4796 (21.8)	11416 (21.0)	3094 (19.9)	8312 (17.7)	2878 (18.4)	7196 (19.8)	2940 (20.8)	5442 (20.5)	46074 (19.9)
23–26	5023 (22.9)	12796 (23.6)	3476 (22.3)	9657 (20.6)	3254 (20.8)	7621 (21.0)	2909 (20.5)	5699 (21.5)	50435 (21.8)
Number of years of school education									
None	270 (1.2)	661 (1.2)	198 (1.3)	645 (1.4)	208 (1.3)	582 (1.6)	249 (1.8)	460 (1.7)	3273 (1.4)
1–6	18697 (85.4)	49305 (91.0)	14002 (90.3)	40500 (86.7)	14168 (90.4)	31879 (87.7)	12194 (86.1)	22528 (85.1)	203273 (88.0)
7–12	2925 (13.4)	4204 (7.8)	1287 (8.3)	5485 (11.7)	1270 (8.1)	3865 (10.6)	1702 (12.0)	3395 (12.8)	24133 (10.5)
>12	1 (0.0)	16 (0.0)	11 (0.1)	86 (0.2)	27 (0.2)	16 (0.1)	18 (0.1)	83 (0.3)	258 (0.1)
Marital status									
Single	10649 (69.2)	28741 (74.3)	7631 (72.9)	22260 (72.1)	7623 (72.9)	18990 (75.4)	9611 (79.2)	17849 (78.3)	123354 (74.3)
Married	4455 (29.0)	9447 (24.4)	2710 (25.9)	8127 (26.3)	2662 (25.5)	5816 (23.1)	2368 (19.5)	4646 (20.4)	40231 (24.2)
Other	283 (1.8)	473 (1.2)	132 (1.3)	485 (1.6)	165 (1.6)	374 (1.5)	161 (1.3)	313 (1.4)	2386 (1.4)
Area of residence									
Urban	11809 (53.8)	33214 (61.1)	9000 (57.9)	27503 (58.6)	9110 (58.1)	20926 (57.6)	7784 (55.0)	14423 (54.4)	133769 (57.8)
Rural	10149 (46.2)	21115 (38.9)	6556 (42.1)	19404 (41.4)	6563 (41.9)	15432 (42.4)	6379 (45.0)	12092 (45.6)	97690 (42.2)

Table 2 shows the results of APC modeling. The coefficient for age in current smokers increased sharply in both males and females. The coefficient for age in former smokers decreased slightly in both males and females, though for female smokers this was not statistically significant.

**Table 2:** Coefficient of current and former smoking from the age-period-cohort model

***Variable***	***Males***		***Females***	
	***Coefficient (95% CI)***		***Coefficient (95% CI)***	
	***Current***	***Former***	***Current***	***Former***
Age (years)				
11–14	Reference	Reference	Reference	Reference
15–18	3.33 (3.14, 3.52)	−0.71 (−1.16, −0.26)	1.39 (0.73, 2.05)	−0.58 (−1.87, 0.70)
19–22	4.36 (4.17, 4.55)	−0.63 (−1.08, −0.18)	2.72 (2.06, 3.38)	−0.88 (−2.15, 0.39)
23–26	4.56 (4.37, 4.74)	−0.37 (−0.82, 0.07)	2.95 (2.29, 3.61)	−0.87 (−2.10, 0.37)
Period				
1996	0.14 (0.08, 0.20)	−0.32 (−0.64, 0.01)	0.37 (0.04, 0.71)	−0.07 (−0.92, 0.79)
2001	Reference	Reference	Reference	Reference
2004	0.02 (−0.04, 0.08)	0.61 (0.36, 0.85)	−0.10 (−0.48, 0.27)	0.62 (−0.06, 1.29)
2007	0.06 (0.01, 0.12)	0.74 (0.51, 0.98)	−0.44 (−0.81, −0.07)	0.42 (−0.31, 1.16)
2009	0.04 (−0.04, 0.11)	1.50 (1.26, 1.74)	0.19 (−0.26, 0.65)	0.53 (−0.23, 1.29)
2011	0.04 (−0.03, 0.11)	0.65 (0.42, 0.89)	−0.11 (−0.57, 0.35)	0.25 (−0.55, 1.05)
2013	−0.02 (−0.10, 0.05)	1.09 (0.87, 1.31)	0.07 (−0.40, 0.53)	0.76 (−0.02, 1.53)
2015	−0.05 (−0.12, 0.01)	0.91 (0.71, 1.11)	−0.28 (−0.68, 0.13)	1.37 (0.73, 2.00)
Birth cohort^*^				
1969–1972	0.03 (−0.05, 0.11)	0.23 (−0.16, 0.61)	−0.10 (−0.53, 0.33)	0.10 (−1.01, 1.21)
1973–1976	Reference	Reference	Reference	Reference
1977–1980	−0.08 (−0.13, −0.04)	−0.21 (−0.43, 0.01)	−0.12 (−0.42, 0.18)	0.28 (−0.38, 0.94)
1981–1984	−0.23 (−0.29, −0.17)	−0.19 (−0.43, 0.05)	0.18 (−0.18, 0.54)	−0.35 (−1.12, 0.43)
1985–1988	−0.17 (−0.25, −0.10)	−0.32 (−0.56, −0.07)	−0.16 (−0.60, 0.28)	0.37 (−0.36, 1.11)
1989–1992	−0.14 (−0.21, −0.07)	−0.25 (−0.47, −0.02)	−0.25 (−0.71, 0.22)	0.62 (−0.14, 1.39)
1993–1996	−0.11 (−0.18, −0.04)	−0.16 (−0.37, 0.05)	0.16 (−0.30, 0.62)	−0.13 (−0.89, 0.63)
1997–2000	−0.04 (−0.11, 0.02)	−0.03 (−0.20, 0.15)	0.16 (−0.26, 0.59)	−0.17 (−0.72, 0.37)

With regard to a period effect, the coefficient for currently smoking males increased slightly from 2001 to 2011 but then decreased between 2013 and 2015. In females, the coefficient fluctuated over time and was rarely statistically significant. An increase in the number of former smokers was detected.

When the data was analyzed for a cohort effect, the coefficient for the younger cohorts of current smokers was lower than the oldest cohort, especially in male smokers. Among former smokers, younger cohorts had a lower coefficient than the oldest cohort in both males and females. However, there was fluctuation in former female smokers.

## Discussion

Our study showed national trends in smoking (both current and former smoking) in the 20-year period between 1996 and 2015. The results chart the prevalence of community-based smoking behavior and the relationships between prevalence and age, survey year, and birth cohort among youths in Thailand. These national survey estimates should have reasonable validity for investigating secular trends in smoking in the community. The results show the age effect, period effect, and cohort effect on the prevalence of smoking among Thailand’s youths.

Since the 1970s, Thailand has implemented many tobacco control programs at the national and community level. The prevalence of current smoking among youths decreased over time from 1996 to 2015, and the prevalence of former smoking decreased in both males and females. The declining trend in current smoking is likely to be due to the effect of tobacco control programs. Even though the prevalence of smoking slightly declined over time, the results highlight the high prevalence of smoking among males and low prevalence among females. Our results contrast with a previous study (21), which found that the number of 13- to 17-year-old students smoking increased between 2005 and 2008. This disagreement is probably due to differences in study sampling and methods of data collection. In the Health and Welfare Surveys we used, information on youths was obtained by proxies, whereas Sirirassamee and Sirirassamee interviewed students directly at school (21). The decrease in current rates of smoking among youths confirms the period effect results obtained from APC modeling.

The prevalence of current smoking increased, while that of former smoking decreased. Since 1992, Thailand has prohibited shops from selling tobacco products to underage customers (i.e. aged less than 18 yr). Our results showed that the prevalence of smoking is low but not negligible among this group. Successful promotion strategies by tobacco companies or perhaps weak enforcement laws may have had an impact on the smoking behavior of this group of youths. In Thailand, tobacco retailers do not usually ask for personal identification or require customers to provide proof of age when purchasing cigarette products at their shops. Our results are similar to those who found that the rate ratio of current smoking increases with age (22).

Our results also showed that younger cohorts tend to smoke less than the oldest cohort, probably because most tobacco control programs in Thailand were launched in or after 2001. Adolescents born between 1985 and 1988 would have been 13 to 16 yr old in 2001 and would have had more exposure to antismoking programs. This finding is in agreement with a previous report by our group showing that younger cohorts are less likely to smoke (23).

A major strength of the current study is that data were obtained from nationally representative surveys conducted using similar methods, so results are likely to be representative of the whole country. One limitation is that, with the Health and Welfare Survey, only the head of the family gives information on the smoking behavior of each family member and prevalence might, therefore, have been underestimated. Moreover, the intervals between surveys were not consistent and this may have affected the estimates obtained using the APC model.

## Conclusion

Although the prevalence of current smoking among Thailand’s youths declined between 1999 and 2015, further reductions are needed. Our findings highlight the necessity of reorienting smoking prevention campaigns towards this population group.

## Ethical considerations

Ethical issues (Including plagiarism, informed consent, misconduct, data fabrication and/or falsification, double publication and/or submission, redundancy, etc.) have been completely observed by the authors.
